# An Ecologic Study of the Association between 1,3-Dichloropropene and Pancreatic Cancer

**DOI:** 10.3390/cancers15010150

**Published:** 2022-12-27

**Authors:** Gerald McGwin, Russell L Griffin

**Affiliations:** Department of Epidemiology, University of Alabama at Birmingham, Birmingham, AL 35294, USA

**Keywords:** dichloropropene, pancreas, cancer, organochlorine

## Abstract

**Simple Summary:**

Prior research has reported an association between 1,3-dichloropropene exposure and pancreatic cancer but was limited by data from a single state in the United States. The current study utilized an ecologic study design to examine the association between 1,3-dichloropropene and pancreatic cancer mortality rates using national-level United states data. Data on 1,3-dichloropropene use was collected from the U.S. Geologic Survey’s National Water-Quality Assessment Project, and pancreatic cancer mortality data was derived from compressed mortality files provided by the Centers for Disease Control and Prevention. Overall, no significant association was observed between 1,3-dichloropropene use and pancreatic cancer mortality rate; however, a significantly increased mortality rate was observed for the highest two quartiles of 1,3-dichloropropene use in states that reported use to the National Water-Quality Assessment Project for at least 20 years.

**Abstract:**

Background: 1,3-Dichloropropene (1,3-D) is a soil fumigant that is used to protect fruit, vegetable, field, tree, and vine crops from nematode infestation and soil borne diseases. It is a commonly use pesticide, is applied by either direct injection into the soil or drip irrigation and is highlight volatile. Though currently classified as “Suggestive Evidence of Carcinogenic Potential”, the literature in animal-based studies has inconsistent results and there is limited research among a human population with one study only among the California population. The purpose of the current analysis is to conduct a state-level analysis of the association between 1,3-D and pancreatic cancer mortality. Methods: Data for this ecological study were derived from death certificate data (for pancreatic mortality) from 1999 to 2020 and United States Geologic Survey National Water-Quality Assessment project for years 1992–2016 (1,3-D use). A negative binomial regression adjusted for selected lifestyle risk factors of pancreatic cancer (i.e., obesity, alcohol use, and smoking prevalence) estimated rate ratios (RRs) and associated 95% confidence intervals (CIs)for the association between 1,3-D quartiles and pancreatic cancer mortality rate. Models lagged in five-year increments to account for the induction period of pancreatic cancer. Results: Overall, there was no association between 1,3-D quartile and pancreatic cancer mortality rate; however, limiting the analyses to states reporting 1,3-D use for at least 20 years, the highest quartile of 1,3-D use was associated with an 11% increase in the pancreatic cancer mortality rate in the five-year lagged model (RR 1.11, 95% CI 1.06–1.16). This association was consistent across the other lag periods. Conclusions: Accounting for lifestyle factors associated with pancreatic cancer risk, there is a significantly increase rate of pancreatic cancer mortality among states that have the highest quartile of 1,3-D use and have been using 1,3-D for a long-term period.

## 1. Introduction

1,3-Dichloropropene (1,3-D) is a pre-plant soil fumigant used to control nematodes and soil borne diseases for a wide variety of fruit, vegetable, field, tree and vine crops. It is the eight most used pesticide in the United States with approximately 25 million pounds applied annually [1]. 1,3-D is applied via direct injection into soil, drip irrigation or broadcasting; it is highly volatile. The primary route of exposure is inhalation though oral exposure occurs via 1,3-D contaminated food and drinking water, though such exposure are thought to be inconsequential. For applicators, acute exposure, regardless of the route, is mitigated via the use of personal protective equipment. Buffer zones and tarping are used to minimize community exposures during periods of application. There are proposals to extend buffer zones and the length of time the treated ground must be tarped to further limit community exposure.

The United States Environmental Protection Agency (EPA) classified 1,3-D as “Likely to be Carcinogenic to Humans” until 2019 when the classification was changed to “Suggestive Evidence of Carcinogenic Potential”. This change was partly based on conjecture that the increased cancer risks observed in early studies were due to a stabilizer ingredient (i.e., epichlorohydrin) rather than 1,3-D itself. Subsequent animal studies using the modern form of 1,3-D have yielded inconsistent results with respect to carcinogenicity, which some studies reporting an increased risk for liver and lung tumors in some species at certain doses [2,3]. To date there has been only one published epidemiologic study regarding the association between 1,3-D exposure and cancer. Clary et al. (2003) conducted an ecological study in three agricultural counties in California, specifically estimating the association between a specific organochlorines and pancreatic cancer mortality [4]. The results indicated that zip codes with higher levels of 1,3-D application had an increased pancreatic cancer mortality rates, specifically for those deaths among persons residing in the counties of interest for at least 20 years.

More broadly, there are a number of studies regarding the association between organochlorine or pesticide exposure generally, typically in occupational settings, and pancreatic cancer. The majority of studies among a human population report positive associations but are limited by the imprecise association estimated owing to the small number of pancreatic cancer cases in any given study. Combined with the observation that that studies reporting an increased risk of other cancers are largely limited to animal studies, the scope of the current analysis is narrowed to focus on pancreatic cancer with an objective to expand upon the previously reported association with pancreatic cancer [4] by performing an ecological study design to explore the relationship between county- and state-level 1,3-D exposure and pancreatic cancer mortality in the conterminous United States.

## 2. Materials and Methods

### 2.1. Data Sources

Data on annual estimated use of 1,3-dichloropropene was derived from the United States Geologic Survey (USGS) National Water-Quality Assessment (NAWQA) project for years 1992–2016. Details of the estimation methodology have been published elsewhere [5], but in short the USGS uses data from pesticide-by-crop use, harvested crop acreage as reported by the crop reporting districts (CRDs) throughout the United States (from both the Census of Agriculture and the National Agricultural Statistics Service’s annual crop harvesting surveys), and geospatial data to make inferences of estimated crop acreage and pesticide use at the county level. A sample of CRDs was surveyed to provide estimated pesticide-by-crop usage by dividing the estimated pesticide use by harvest crop acreage. For those CRDs not surveyed, pesticide use was estimated based on the median values of pesticide use of neighboring surveyed CRDs. Within the NAWQA data, there are CRDs that have a null value for estimated pesticide use due to inadequate farm survey data for a given watershed [6]. Data on annual pancreatic mortality rates were derived from death certificate data provided by the National Center for Health Statistics and accessed from the Center for Disease Control and Prevention’s Wide-ranging ONline Data for Epidemiologic Research (WONDER) online data querying system. For purposes of the current analysis, mortality data was limited to 1999 to 2020, the most recent non-provisional data available. State-level population counts were derived from U.S. Census intercensal population estimates, and state-level land area data was obtained from the U.S. Census Bureau.

### 2.2. Variable Definitions

The exposure of interest was state-level use of 1,3-D defined as the sum of kilograms of 1,3-D use across all reported crops divided by the square mileage (sq mile) of land area for the respective state. A five-year moving average of the kg/sq mile was used to in the current analysis to minimize the effect of annual fluctuations in use; for purposes of the current analysis, 1,3-D use was categorized into the following quartiles: ≤0.40 kg/sq mi, 0.41–2.2 kg/sq mile, 2.3–7.9 kg/sq mile, and ≥8 kg/sq mile. If a state was missing data for a given year (e.g., Delaware for years other than 1992, 1993, and 1998), the estimated 1,3-D use for the state for that year was assumed to be zero. Each state was defined by how many years of 1,3-D use was reported with a max of 25 years and using categories of 0–9 years, 10–14 years, 15–19 years, 20–24 years, and 25 years. The main outcome of interest was pancreatic cancer mortality, defined as a death in which pancreatic cancer was listed as an underlying or contributing cause. Based on a recent review article [7], BRFSS data was queried [8] to collect state-level data regarding the statewide prevalence of obesity, alcohol use (drank alcohol within the past 30 days), and current smokers. Of note, BRFSS data were not available for the entire study period; as a result, the year 2011 was chosen as it was the median year of the 1999 to 2020 pancreatic case period.

### 2.3. Statistical Analysis

**Figure 1:**
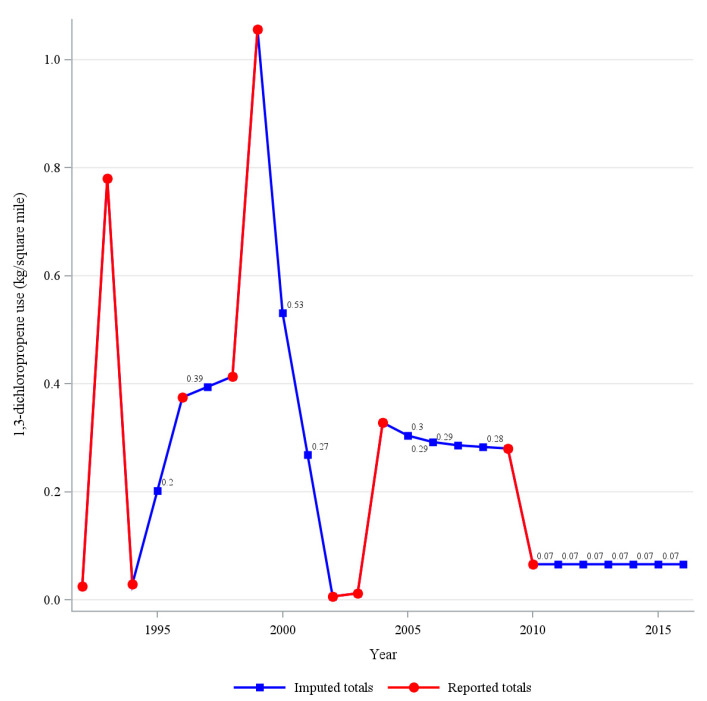
Reported and imputed values of 1,3-dichloropropene for Pennsylvania.

A choropleth map of combined quartile of 1,3-D use and pancreatic cancer mortality rate was created to provide a visual analysis of geographic areas where the pesticide use and mortality rates were highest. A Kruskal–Wallis test with a Dwass, Steel, Critchlow-Fligner Method for pairwise comparisons was used to compare the distribution of 1,3-D use by categories of years reported for each lagged analysis exposure (i.e., 5-, 10-, 15-, and 20-year lagged exposed). A negative binomial model adjusted for state-level prevalence of obesity, alcohol use, and smoking was used to estimate rate ratios (RRs) and associated 95% confidence intervals (CIs) for the association between 1,3 dichloropropene use quartile and pancreatic cancer mortality rate using state population as the model offset. Models were created using 5-, 10-, 15-, and 20-year lag periods to account for induction period of pancreatic cancer, which is not a known number but reported to be between 10 and 20 years. In a sensitivity analysis, models were limited to states with 20 or more years of reported 1,3-D use. Two sets of models were used: one in which missing values of 1,3-D use due to inadequate farm survey data were assumed to be zero (i.e., complete data) and another model in which values of missing data were imputed based upon the values of the immediate preceding and proceeding non-missing values. The imputation was performed by imputing missing values as the average between the two non-missing values, thereby keeping the imputed values within the trends between two non-missing values (see Figure 1 for an example).

## 3. Results

**Figure 2:**
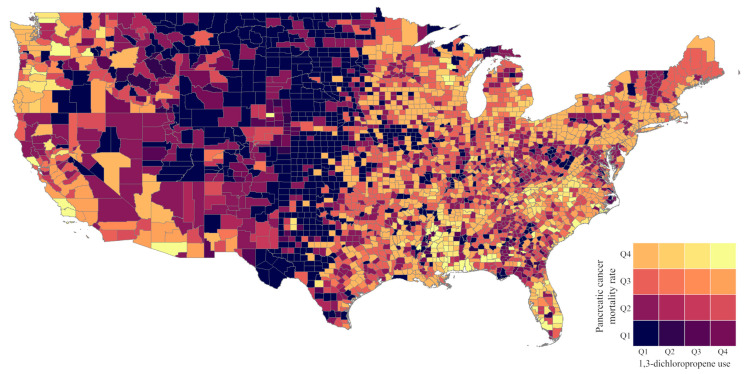
Choropleth map of combined 1,3-dichloropropene use and pancreatic cancer mortality rate by county.

The counties with the highest combined quartiles of 1,3-D use and pancreatic cancer mortality rate were most located in North Carolina, South Carolina, Florida, Alabama, and Mississippi though a few were in California, Oregon, and Washington state (Figure 2). Overall the median use of 1,3-D ranged between 2.0 (IQR 0.4–7.9) to 2.5 (IQR 0.5–7.9) kg/square mile depending on the year lag with the 20-year lag having the highest median (Table 1). The distribution of amount of 1,3-D was significantly different (*p* < 0.0001) among years-reported category for all lagged exposures. In pairwise comparisons, for each lagged exposure the significant difference was driven by a higher reported use among states that reported 1,3-D use for either 20–24 years or 25 years. There was no difference in the distribution of reported use among states reporting 0–9, 10–14, or 15–19 years. The distribution of the pancreatic cancer mortality rate was differential by the number of years of reported 1,3-D use (Kruskal–Wallis *p* < 0.0001) with the differences driven by states with 20–24 or 25 years of use having a distribution more towards the lower end of pancreatic cancer mortality (Table 2).

By years of usage reported, median use was highest among the 20–24 and 25-year categories and lowest among the 10–14 and 15–19 year categories. In regression models, each of the potential confounders were significantly associated with the pancreatic cancer mortality rate with similar associations observed for each increase in the median percent of the population that was obese (RR 1.019 (95% CI 1.013–1.025), used alcohol (RR 1.007, 95% CI 1.005–1.009), or used tobacco (RR 1.017, 95% CI 1.012–1.022). Regarding exposure to 1,3-D, in general there was no association between 1,3-D exposure quartile and pancreatic cancer mortality rate (Table 3); however, when limiting data to states which reported 1,3-D use for 20–25 years of the study period, higher quartile of use was associated with an increased rate of pancreatic cancer-related mortality. Specifically, the highest quartile of use, when compared to states with the lowest quartile of use, had an 11% higher mortality rate in five-year lagged models (RR 1.11, 95% CI 1.06–1.16). This association was similar across lagged models with slightly weaker associations observed for the highest quartile of use for the 15-year (RR 1.08, 95% CI 1.02–1.14) and 20-year (RR 1.09, 95% CI 1.02–1.16) lagged models. For all lagged analyses, there was a significant (*p* < 0.0001) test of linear trend across quartile categories. Similar associations were observed when using the imputed data.

## 4. Discussion

Among states with consistently reported data on 1,3-D application, there is a statistically significant positive association between pancreatic cancer mortality and the density of 1,3-D application. The magnitude of the association remained stable with longer lags in the exposure. When states with inconsistent 1,3-D application data were included in the analysis, the associations were null. This is likely due to the fact that the assumption that missing 1,3-D use values denoted no use resulted in a misclassification bias towards the null as the quantification of use was underestimated for some states.

To date there has only been one other peer-reviewed study that has estimated in the association between 1,3-D exposure and pancreatic cancer. Clary et al. conducted an ecologic study comparing zip code level organochlorine use and pancreatic cancer mortality in three California counties (4). The results indicated that pancreatic cancer mortality was elevated in zip codes with the highest use of 1,3-D, among other pesticides. A case-report describing a fatal 1,3-D intoxication suggested that damage to the pancreas was caused by the chemical [9]. There have been, however, a number of studies examining the association between pancreatic cancer and pesticide exposure generally, as well as organochlorine exposure specifically. Over the past several decades, a number of studies have explored the association between pancreatic cancer and occupational exposures, including pesticides. In some of these studies, pesticide exposure was inferred based upon occupation (e.g., farming) whereas in others, study participants provided information about work-related pesticide exposures. The results of these studies are largely equivocal with some reporting excess risks whereas others do not [10]. This inconsistency has been partly attributed to the small number of pancreatic cancer cases in some studies; misclassification of pesticide exposure likely also played a role. Interestingly, studies that elicited information directly from study participants regarding pesticide exposure tended to report positive associations. In a case–control study examining the epidemiology of pancreatic cancer in Egypt, cases had a 2.6-fold increased odds of exposure to pesticides [11]. Among a cohort of agricultural workers in Iowa and North Carolina, self-reported organochlorine exposure was associated with a non-significantly increased risk of pancreatic cancer [12]. A related study of the spouses of the agricultural workers, a similar non-significantly increased risk of pancreatic cancer was observed [13]. Though fewer in number, there have also been studies examining non-occupational pesticide exposures. Antwi et al. (2015) reported that self-reported regular exposure to pesticides was associated with a significantly increased odds of pancreatic cancer; the association was limited to those who worked in indoor environments [14]. Fryzek et al. (1997) observed a non-statistically significant increased odds ratios for self-reported exposure to organochlorines [15]. Finally, at least two studies have examined the relationship between organochlorine exposure biomarkers and pancreatic cancer risk, with conflicting results [16,17]. In both studies, biomarkers for 1,3-D exposure were not quantified, the focus being on other organochlorines such as DDT and PCBs.

Animal studies provide little additional context for the association between 1,3-D and pancreatic cancer. While studies have shown an increased occurrence of liver, stomach bladder and lung tumors, studies specific to pancreatic cancer are absent [2,3]. Though, to knowledge, there is no literature on the biological mechanism of 1,3-D and pancreatic cancer, one potential mechanism could be through K-ras mutations. These mutations, present in approximately 85% of pancreatic cancers [18], are reported to be involved with the growth and maintenance of tumors (not specifically pancreatic tumors) and to be a potential driving factor of the lower survival rate of pancreatic cancer [19]. Further, studies have reported that organochlorines such as 1,1-dichloro-2,2-bis(p-chlorophenyl) ethylene (p,p′-DDE) (p,p′-DDE) and 1,1,1-trichloro-2,2-bis(p-chlorophenyl)-ethane (p,p′-DDT) are associated with increased odds K-ras mutations [20,21], providing a potential mechanism of action for organochlorines like 1,3-D on the development of exocrine pancreatic cancer. Another potential mechanism includes the metabolism of 1,3-D in the body. One proposed metabolic process of 1,3-D involves epoxidation in the reaction with cytochrome P450, with higher exposure levels of 1,3-D being associated with the P450-dependent process [22]. The action of cytochrome P450 is associated with the production of reactive oxygen species [23], which could lead to oxidative stress, which, is associated with cellular damage, inflammation, and, particularly, oncogenesis [24,25].

## 5. Limitations

The results of the current study should be interpreted in light of certain strengths and limitations. The current study was strengthened by the use of multiple decades of data, allowing for the examination of lag periods to attempt to account for the induction period between 1,3-D use exposure and pancreatic cancer. Given the ecologic study design, information regarding pancreatic cancer and 1,3-D is in the aggregate and thus individualized exposures could not be quantified. Additionally, given the rarity of pancreatic cancer, in order to obtain stable measures of association the unit of analysis was the state. 1,3-D use is localized to the agricultural regions of a state and thus state-level metrics are likely to underestimate and overestimate true individual exposure levels. The outcome for this study was pancreatic cancer mortality; however, given the relatively high case-fatality rate, this is a reasonable proxy for incidence. In the current analysis, missing values of 1,3-D use due to inadequate farm survey data were assumed to be zero; however, this introduces a misclassification bias towards the null as it is possible those missing data could have actually used 1,3-D on the crops for that year. This could explain the near-null protective effects observed in the all-state analysis; by limiting the analysis to states that had at least 20 years of reported estimated 1,3-D use, the potential for bias was minimized. We controlled our associations for lifestyle factors such as alcohol use, obesity, and smoking; however, the associations are provided by the BRFSS as the median value. As a result, it is possible that the reported associations may be continued to be biased by residual confounding of these confounders as well as other unmeasured confounders.

## 6. Conclusions

The carcinogenic potential for 1,3-D was recently downgraded by the EPA from “likely to be carcinogenic” to “suggestive evidence of carcinogenic potential”; this change was supported by some and decried by others. While any such change would elicit supporters and detractors, in the case of 1,3-D the impetus for it and the associated debate reflects the lack of research on the topic. The broader literature on pesticide and organochlorine exposure provides limited insight. The current study is only the second to specifically address the association between 1,3-D and pancreatic cancer, both using an ecologic study design and both reporting weak but positive associations. Such findings amidst the limitations of the study design should not be ignored and warrant more rigorous study.

## Figures and Tables

**Table 1:** Median and interquartile range of 1,3 dichloropropene use by number of years of use data reported to the USGS, 1992–2016.

Years 1,3-D Use Reported		Median (kg/sq Mile)	25th Percentile (kg/sq Mile)	75th Percentile (kg/sq Mile)
Overall	5-year lag (*n* = 646)	2.12	0.40	7.94
	10-year lag (*n* = 583)	2.03	0.41	7.63
	15-year lag (*n* = 425)	2.17	0.47	7.30
	20-year lag (*n* = 271)	2.52	0.47	7.88
				
0–9	5-year lag (*n* = 36)	0.99	0.09	6.23
	10-year lag (*n* = 43)	1.01	0.12	7.57
	15-year lag (*n* = 35)	0.96	0.12	7.57
	20-year lag (*n* = 26)	0.42	0.05	7.57
				
10–14	5-year lag (*n* = 48)	0.27	0.08	0.72
	10-year lag (*n* = 51)	0.27	0.11	0.84
	15-year lag (*n* = 37)	0.30	0.14	1.47
	20-year lag (*n* = 26)	0.29	0.14	0.84
				
15–19	5-year lag (*n* = 75)	0.42	0.09	1.64
	10-year lag (*n* = 68)	0.51	0.12	1.78
	15-year lag (*n* = 44)	0.71	0.12	2.85
	20-year lag (*n* = 20)	1.08	0.09	5.80
				
20–24	5-year lag (*n* = 113)	2.02	0.70	2.83
	10-year lag (*n* = 98)	2.03	0.75	4.04
	15-year lag (*n* = 71)	1.97	0.77	2.59
	20-year lag (*n* = 46)	1.84	0.93	3.77
				
25	5-year lag (*n* = 374)	4.77	0.68	13.32
	10-year lag (*n* = 323)	4.88	0.74	12.54
	15-year lag (*n* = 238)	4.55	0.80	12.11
	20-year lag (*n* = 153)	4.33	1.12	11.68

**Table 2:** Median and interquartile range of pancreatic cancer-related mortality rate by number of years of use data reported to the USGS, 1999–2020.

Years 1,3-D Use Reported	Median (per 100,000 Persons)	25th Percentile (per 100,000 Persons)	75th Percentile (per 100,000 Persons)
Overall	12.75	11.32	14.27
Years of Use Reported			
0–9	13.50	12.43	14.85
10–14	13.68	12.09	15.21
15–19	12.62	11.59	14.16
20–24	12.34	11.28	14.25
25	11.61	10.23	13.24

**Table 3:** Rate ratios * (RRs) and associated 95% confidence intervals (CIs) for the association between 1,3 dichloropropene (1,3-D) use and pancreatic cancer-related mortality.

1,3-D Use Quartiles-	All States		States Reporting 20+ Years of 1,3-D Use	
	Complete ^†^ Data	Imputed Data	Complete Data	Imputed Data
	RR (95% CI)	RR (95% CI)	RR (95% CI)	RR (95% CI)
5-YEAR LAG				
≤0.40 kg/sq mi	Referent	Referent	Referent	Referent
0.41–2.2 kg/sq mile	0.97 (0.94–1.00)	0.98 (0.95–1.00)	0.98 (0.93–1.03)	0.99 (0.94–1.03)
2.3–7.9 kg/sq mile	1.00 (0.97–1.03)	0.97 (0.95–1.00)	1.03 (0.98–1.09)	1.05 (1.00–1.10)
≥8 kg/sq mile	1.06 (1.02–1.09)	1.02 (0.99–1.05)	1.10 (1.05–1.15)	1.11 (1.06–1.16)
10-YEAR LAG				
≤0.40 kg/sq mi	Referent	Referent	Referent	Referent
0.41–2.2 kg/sq mile	0.96 (0.93–0.99)	0.99 (0.96–1.01)	0.99 (0.94–1.04)	0.99 (0.95–1.04)
2.3–7.9 kg/sq mile	0.96 (0.93–1.00)	0.96 (0.94–0.99)	1.02 (0.97–1.08)	1.03 (0.98–1.08)
≥8 kg/sq mile	1.03 (0.99–1.07)	1.02 (0.99–1.05)	1.11 (1.05–1.16)	1.11 (1.06–1.16)
15-YEAR LAG				
≤0.40 kg/sq mi	Referent	Referent	Referent	Referent
0.41–2.2 kg/sq mile	0.96 (0.93–1.00)	0.98 (0.95–1.01)	0.98 (0.92–1.04)	0.98 (0.93–1.04)
2.3–7.9 kg/sq mile	0.98 (0.95–1.02)	0.97 (0.95–1.00)	1.03 (0.98–1.10)	1.04 (0.98–1.09)
≥8 kg/sq mile	1.01 (0.97–1.05)	1.00 (0.97–1.04)	1.07 (1.01–1.14)	1.08 (1.02–1.14)
20-YEAR LAG				
≤0.40 kg/sq mi	Referent	Referent	Referent	Referent
0.41–2.2 kg/sq mile	0.92 (0.88–0.96)	0.94 (0.91–0.97)	0.98 (0.91–1.05)	0.98 (0.92–1.04)
2.3–7.9 kg/sq mile	0.98 (0.94–1.03)	0.98 (0.95–1.01)	1.08 (1.00–1.15)	1.07 (1.00–1.14)
≥8 kg/sq mile	1.00 (0.96–1.04)	0.99 (0.95–1.02)	1.09 (1.02–1.17)	1.09 (1.02–1.16)

* Estimated from a negative binomial regression adjusted for (based on 2011 Behavioral Risk Factors Surveillance Survey data) estimated percent of the state population that was obese, used alcohol within the past 30 days, and was a current smoker; † Complete data assumes missing 1,3-D use values are 0; imputed data estimate a value for missing values based on the immediate preceding and proceeding non-missing 1,3-D use values.

## Data Availability

Data on 1,3-dichloroporopene use in the United States can be access from the United States Geologic Survey’s National Water-Quality Assessment Project at https://water.usgs.gov/nawqa/pnsp/usage/maps/county-level/ (accessed on 13 December 2022). Data on pancreatic cancer mortality in the United States can be accessed from the CDC’s Wide-ranging ONline Data for Epidemiologic Research data system at https://wonder.cdc.gov/ (accessed on 13 December 2022) under the Multiple Causes of Death database.
